# Acute MR-Guided High-Intensity Focused Ultrasound Lesion Assessment Using Diffusion-Weighted Imaging and Histological Analysis

**DOI:** 10.3389/fneur.2019.01069

**Published:** 2019-10-15

**Authors:** Matthew R. Walker, Jidan Zhong, Adam C. Waspe, Thomas Looi, Karolina Piorkowska, Cynthia Hawkins, James M. Drake, Mojgan Hodaie

**Affiliations:** ^1^Institute of Medical Science, University of Toronto, Toronto, ON, Canada; ^2^Division of Brain, Imaging and Behaviour – Systems Neuroscience, Krembil Research Institute, University Health Network, Toronto, ON, Canada; ^3^Centre for Image Guided Innovation and Therapeutic Intervention, Hospital for Sick Children, Toronto, ON, Canada; ^4^Department of Medical Imaging, University of Toronto, Toronto, ON, Canada; ^5^Department of Paediatric Laboratory Medicine, Division of Neuropathology, Hospital for Sick Children, Toronto, ON, Canada; ^6^Division of Neurosurgery, Hospital for Sick Children, Toronto, ON, Canada; ^7^Division of Neurosurgery, Toronto Western Hospital, University Health Network, Toronto, ON, Canada

**Keywords:** focused ultrasound, magnetic resonance-guided focused ultrasound (MRgFUS), diffusion tensor imaging (DTI), diffusion weighted imaging (DWI), tractography

## Abstract

**Objectives:** The application of magnetic resonance-guided focused ultrasound (MRgFUS) for the treatment of neurological conditions has been of increasing interest. Conventional MR imaging can provide structural information about the effect of MRgFUS, where differences in ablated tissue can be seen, but it lacks information about the status of the cellular environment or neural microstructure. We investigate *in vivo* acute changes in water diffusion and white matter tracts in the brain of a piglet model after MRgFUS treatment using diffusion-weighted imaging (DWI) with histological verification of treatment-related changes.

**Methods:** MRgFUS was used to treat the anterior body of the fornix in four piglets. T1 and diffusion-weighted images were collected before and after treatment. Mean diffusion-weighted imaging (MDWI) images were generated to measure lesion volumes via signal intensity thresholds. Histological data were collected for volume comparison and assessment of treatment effect. DWI metric maps of fractional anisotropy (FA), apparent diffusion coefficient (ADC), axial diffusivity (AD), radial diffusivity (RD), and mean diffusivity (MD) were generated for quantitative assessment. Fornix-related fiber tracts were generated before and after treatment for qualitative assessment.

**Results:** The volume of treated tissue measured via MDWI did not differ significantly from histological measurements, and both were significantly larger than the treatment cell volume. Diffusion metrics in the treatment region were significantly decreased following MRgFUS treatment, with the peak change seen at the lesion core and decreasing radially. Histological analysis confirmed an area of coagulative necrosis in the targeted region with sharp demarcation zone with surrounding brain. Tractography from the lesion core and the fornix revealed fiber disruptions following treatment.

**Conclusions:** Diffusion maps and fiber tractography are an effective method for assessing lesion volumes and microstructural changes *in vivo* following MRgFUS treatment. This study demonstrates that DWI has the potential to advance MRgFUS by providing convenient *in vivo* microstructural lesion and fiber tractography assessment after treatment.

## Introduction

Magnetic resonance-guided focused ultrasound (MRgFUS) is a novel thermal ablation method that non-invasively destructs tissues by thermal coagulation (1). The guidance of MR imaging allows therapeutic targets to be accurately identified and treated (2). While MRgFUS has been previously used to treat uterine fibroids, bone metastases, prostate cancer, and liver tumors (2), recent work has focused on the treatment of essential tremor and tremor associated with Parkinson's disease (3–6), neuropathic pain (7), obsessive–compulsive disorder (8), and epilepsy (9), suggesting that MRgFUS is a promising tool for the treatment of brain disorders.

While changes related to treatment can be discerned, current MR sequences, such as T1-weighted imaging, do little to distinguish detailed microstructural changes in the lesioned areas (10). Ablated tissue appears hypointense in T1 images due to vascular coagulation but provides no further information about the cellular environment. Diffusion-weighted imaging (DWI) is sensitive to water diffusion intracellularly and extracellularly (11). Metric information can be derived from DWI by modeling the water diffusion as a tensor. Metrics include axial diffusivity (AD, corresponding to the parallel component of the diffusion tensor), which reflects axonal changes; radial diffusivity (RD, the perpendicular component), which reflects myelination (12); and mean diffusivity (MD, the average of all three tensor components), which is sensitive to cellularity (13). Fractional anisotropy (FA) describes the directionality of water diffusion and reflects the integrity of white matter (14, 15). Apparent diffusion coefficient (ADC), a measure of the movement of water molecules in the tissue (16), indirectly reflects tissue cellularity and the integrity of the cell membrane (17).

Diffusion imaging can also provide three-dimensional visualization of white matter fiber tracts through tractography (15). Crossing fiber regions can be reliably tracked using high angular resolution diffusion imaging (HARDI) (18, 19) and a method of constrained spherical deconvolution proposed by Tournier et al. (20) In this way, diffusion imaging can be used in surgical planning to avoid damaging major tracts connected to an eloquent area, to visualize target regions, or to assess the effectiveness of surgical treatments (21–25).

A recent study of MRgFUS thalamotomy for the treatment of essential tremor examined the relationship between adverse side effects and lesion location and size as revealed by diffusion tractography (26). Others have explored pre-treatment tractography for target determination (27–29). There is growing interest in targeting white matter structures directly to treat obsessive–compulsive disorders and epilepsy (30–32). However, few studies have explored the range of microstructural changes within the brain due to MRgFUS lesioning as revealed by DTI metrics. Therefore, a comprehensive evaluation of diffusion metrics and tractography of white matter of the brain following MRgFUS treatment is needed. For this purpose, the fornix has been chosen as a model white matter structure to be accurately targeted with MRgFUS and examined via DWI.

The aim of this study is the assessment of *in vivo* acute changes in diffusion metrics and fiber tractography in the brain of a piglet model after MRgFUS treatment. Histological examination of the lesion sheds light on the cellular changes after ultrasound, and how this corresponds with imaging findings.

## Materials and Methods

### *In vivo* Piglet Model

These experiments were approved by the Animal Care Committee and Lab Animal Services at the Hospital for Sick Children. This study conforms to the Canadian Council on Animal Care (CCAC).

Four Yorkshire male piglets weighing 5.0–6.7 kg (average weight: 5.8 ± 0.8 kg) were pre-anesthetized with a ketamine (10 mg/kg) solution intramuscularly. The animals were intubated using 2.5% isoflurane and 2 L of oxygen.

Under anesthesia, a vertex craniotomy was performed to create an anterior fontanelle of 5 × 7 cm^2^ after hair removal from the animal's forehead via shaving and commercial depilatory cream. The craniotomy was performed to avoid potential ultrasound field distortion caused by the skull. Specific care was taken during the craniotomy procedure to ensure no accidental cut on the surface vessels underneath the skull. After the cranial window was created, a mixture of degassed, deionized water and ultrasound gel (10:1 water-to-gel ratio) was poured on the brain. The skin flap was carefully folded back on top to avoid trapping air bubbles and then sutured closed.

After the surgery, the animals were transferred for pre-treatment MR imaging on a standard diagnostic table. To ensure the appropriate depth of anesthesia, heart rate and oxygen saturation were monitored and a circulating water blanket was used to maintain core body temperature around 37°C.

### MR Imaging

T1 and diffusion imaging data were collected in the same manner in the prone position on the diagnostic table for both pre- and post-treatment time points. Post-treatment imaging was used to evaluate acute tissue changes following treatment compared to pre-treatment imaging.

Using a Philips Achieva 3T MR scanner (Philips Healthcare, Best, Netherlands), a 32-channel receive-only head coil was placed around the head to provide MR imaging. A three-dimensional T1 magnetization prepared gradient echo (MPRAGE) sequence was used. The T1 acquisition parameters included: repetition time (TR) = 8.15 ms; echo time (TE) = 3.72 ms; flip angle = 8°; matrix = 224 × 224; field of view (FOV) = 224 mm × 224 mm; slice thickness = 1.00 mm; number of signal averages (NSA) = 2; acquisition time = 14 min 35 s. The diffusion-weighted images were collected with a SENSE-single shot spin echo (SE)-echo planar imaging sequence (TR = 5844.97 ms; TE = 105.90 ms; flip angle = 90°; matrix = 128 × 128, FOV = 205 mm × 205 mm; slice thickness = 1.60 mm; SENSE reduction factor = 2; NSA = 2; acquisition time = 29 min 33 s) with a *b* value of 800 s/mm^2^ along 128 directions. A baseline image with *b* = 0 s/mm^2^ was acquired with both forward and reverse phase encoding directions for post-processing susceptibility distortion corrections.

### MRgFUS Imaging and Treatment

Imaging and treatment were performed using a clinical MRgFUS system (Sonalleve, Profound Medical, Toronto, Canada) integrated with the MR scanner. After the pre-treatment imaging, the animal was transferred to the MRgFUS table.

**Figure 1:**
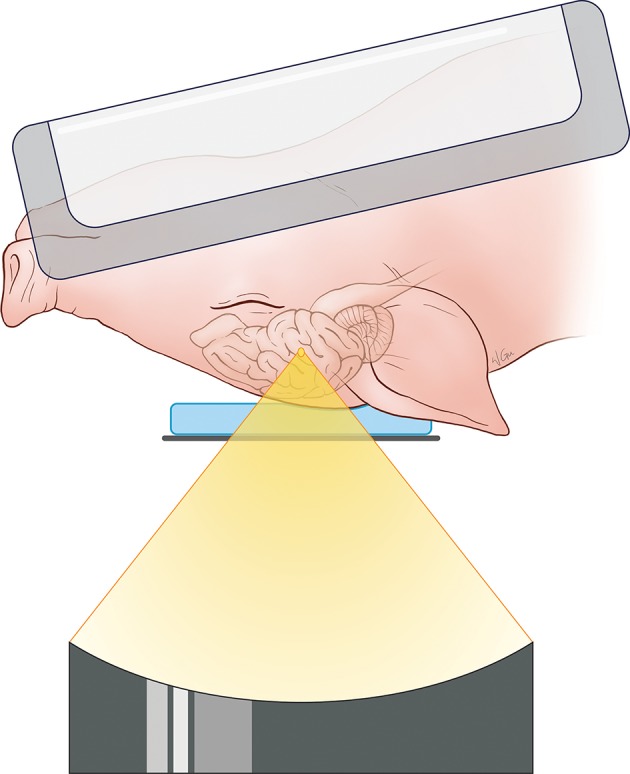
Schematic illustration of animal positioning during MRgFUS treatment.

For the targeting and sonication of the anterior body of the fornix, the animal's head was placed above the ultrasound transducer on the MRgFUS table in a supine position and feet first. A 15-mm thick gel pad (Aquaflex, Parker Laboratories, New Jersey, USA) overlaid with degassed ultrasound gel was placed beneath the head for acoustic coupling with the ultrasound transducer and treatment window. MR signal was acquired with a two-channel SENSE Flex-M coil positioned around the head along with a single receive coil built into the table of the MRgFUS system. A schematic illustration of the animal positioning during treatment is found in Figure 1.

Once the animal was positioned on the MRgFUS system, images were collected to confirm there were no air bubbles at the interface between the brain and the gel pad that might interfere with ultrasound beam propagation.

The location of the fornix was identified at the mid-sagittal plane, anterior to the thalamus. The treatment targets were positioned on sagittal images using the MRgFUS planning software along the anterior body of the fornix with a cross-sectional diameter of 4 mm and a length of 10 mm (volume = 83.78 mm^3^). We applied repeated sonications with increasing power to reach a temperature above 60°C to ensure maximal tissue disruption, based on the method described by Jeanmonod (7). Three to four sonications from 40 to 80 W were performed on the target to reach a peak ablation temperature >60°C. No more sonications were performed after this temperature threshold was attained. Tissue temperatures were monitored in real time via proton resonance frequency shift MR thermometry sequences applied simultaneously with sonication. This thermometry measurement is based on the principle that the proton resonant frequency is dependent on the sample temperature, where changes in temperature result in an image phase shift (33). The thermal distribution is measured relative to the temperature of the subject, which is determined via rectal temperature probe. Each sonication had a duration of 20 s for most of the pigs with the exception being the first animal where the durations were 45, 20, and 15 s. Between each sonication, a 5-min pause was implemented to allow the tissue to cool down. A frequency of 1.2 MHz was used for all sonications. Before each therapeutic session, one to two test sonications at 10 W and 20 s were performed for calibration of the ultrasound focus location. Detailed experimental parameters, including treatment cell sizes, acoustic power applied, and peak temperature reached are listed in Table 1.

**Table 1:** Pig weight, treatment parameters, and lesion measurements from histological and mean diffusion-weighted imaging (MDWI) data.

Pig ID	1	2	3	4
Weight (kg)	5.0	6.7	5.3	6.3
Sonication power (W)	40, 60, 80	40, 60, 70, 80	40, 60, 80	40, 60, 80
Sonication duration (s)	45, 20, 15	20	20	20
Base temperature (°C)	35.5	35.7	35.5	37.7
Final peak temperature (°C)	62.6	64.2	60.8	68.8
Volume of histological lesion (mm^3^)	315	418	297	351
Volume of MDWI lesion core (mm^3^)	302	432	337	319

Following treatment, the animal was repositioned on the diagnostic table for post-treatment MR imaging. Scanning parameters and animal positioning were identical to pre-treatment images.

### Histological Data

Upon completion of post-treatment imaging, the animals were euthanized with an intravenous injection of pentobarbital sodium (120 mg/kg) while under anesthesia. They then underwent cardiac perfusion fixation followed by immersion fixation with formalin for 1 week. Post-fixation, the specimen was sectioned coronally from the two ends of the brain to keep the treated tissue. The treated tissue was then processed with routine histology, sectioned at a 5-micron thickness at the 200-micron level, and stained with hematoxylin and eosin (H&E). Selected slices were stained with luxol fast blue (LFB) to investigate myelin changes.

Lesion boundaries were delineated using the smart segmentation tool in Image Pro Premier (Media Cybernetics, http://www.mediacy.com/). Sample regions of necrosis, edema, and non-treated brain tissue were identified for the classification software to create a segmentation protocol based on slide color, intensity, background, and morphology. This protocol was applied to all histological data to measure lesion areas for each slide. The final volume of treated tissue was calculated as total area per slide multiplied by the 200-micron slide thickness and summed over all slides.

### Image Processing

T1 image processing: The T1-template from Conrad et al. (34) was manually registered to individual T1 images to provide a brain mask using 3D Slicer (version 4.4, https://www.slicer.org/). A linear transformation matrix was generated through this manual registration to each individual T1 image. To calculate the individual brain volume, the brain mask of the T1 template was transformed back to individual image space via the linear transformation generated.

DWI processing: DWI sequences were corrected for susceptibility-induced and motion distortions using Topup (35) and FLIRT in FSL (36). Additionally, the gradient vectors were corrected with the appropriate rotational component of the motion correction to minimize errors in the diffusion weighting (37). The data were resampled for a final voxel size of 1 × 1 × 1 mm^3^. Diffusion tensors were fitted at each voxel to calculate scalar maps: ADC, FA, AD, RD, and MD. Mean diffusion-weighted imaging (MDWI) was calculated as the average of the DWI sequences including the b0 image. Individual brain masks were transformed from T1 space to DWI space through manual registration with FA as the feature using 3D Slicer. The registration was also verified with transformed MDWI images by overlapping on the T1 images. Image transformation matrices are available online (https://www.hodaielab.com/resources.html).

**Figure 2:**
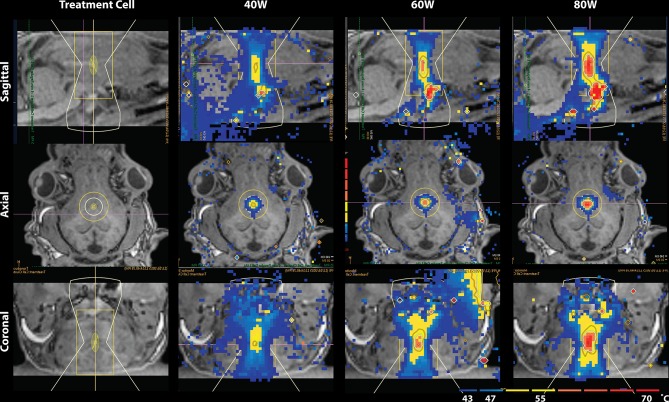
Temperature map during MRgFUS treatment for a single subject in the sagittal view **(Top)**, axial view **(Middle)**, and coronal view **(Bottom)**. Column 1 shows the treatment cell on the fornix. Columns 2–4 show the temperature maps after treatment with 40, 60, and 80W, respectively. In Row 1 of columns 2–4, skull-base heat deposition was present in the far-field region inferior to the target area, close to optic chiasm.

The lesion presented as a global maximum hyperintensity on the post-treatment MDWI image. To delineate the treated volume in this MDWI image, we calculated the percentage of voxels exceeding a specific intensity level compared to the whole brain volume. A cutoff intensity was selected to distinguish the lesion voxels from untreated brain tissue. Due to an inflection point observed in distribution of voxel intensities on the MDWI image, an intensity level with number of voxels smaller than 1.1% over the whole brain volume was taken as the cutoff intensity for the lesion site (Supplementary Figure 1). This contiguous region was then referred to as the lesion boundary. To account for partial volume effects, this volume was eroded by a one voxel layer to isolate the lesion core. To investigate diffusion metric changes on the periphery of the lesion site, a one-voxel dilation was performed on the lesion boundary to get a mask of the outer boundary of the lesion site. Diffusion metrics were computed for the lesion core, lesion boundary, and outer lesion boundary volumes. This erosion and dilation method was used as the temperature contours measured via MR thermometry showed a similar radial layering pattern (Figure 2).

Fiber tracking was performed with the MRtrix software package (Brain Research Institute, Melbourne, Australia, http://www.brain.org.au/software/). The response function for a single fiber population was estimated for voxels with FA > 0.3 using an iterative optimization method (38). The response function was then used for constrained spherical deconvolution to accurately estimate the fiber orientation distribution (FOD) (20). Fibers were then generated with a deterministic tracking algorithm, referred as SD-Stream, that follows the orientation of the nearest FOD peak at each step (39). Fibers were also generated with deterministic tracking based on the single tensor model for a visual comparison. Tracking parameters included a step size of 0.1 mm, a minimum radius of curvature of 1 mm, a FOD cutoff of 0.15, and a minimum length of 5 mm.

### Statistical Analysis

Paired *t* test was performed for normalized white matter metrics from pre- and post-treatment separately in the lesion core, lesion boundary, and outer lesion boundary. Metric normalization was implemented using a reference volume in the splenium of the corpus callosum due to its robust anisotropic diffusion signature. *P*-values were considered significant with <0.05 [false discovery rate (FDR) corrected]. A one-way analysis of variance (ANOVA) was applied for each white matter metric in the lesion core, lesion boundary, and outer lesion boundary, separately for pre- and post-treatment. *Post-hoc* analysis with Tukey's honest significance difference (HSD) test was performed to further investigate the level of difference, considering *P* < 0.05 as significant.

## Results

### MRgFUS Treatment

**Figure 3:**
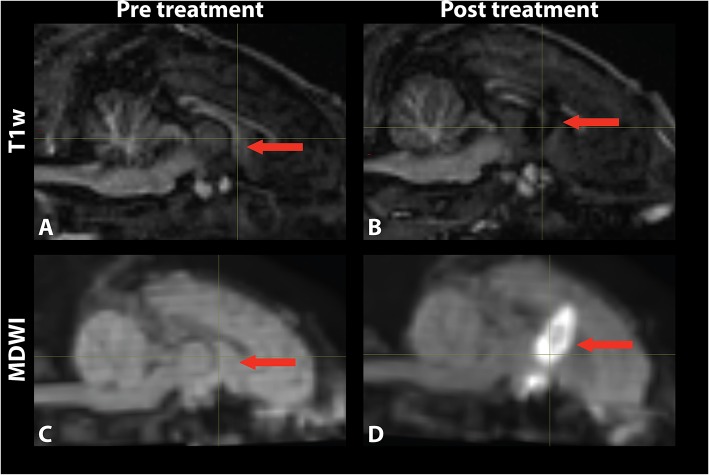
Mid-sagittal **(A,B)** T1-weighted and **(C,D)** MDWI images before and after MRgFUS treatment for a single subject. The target area appeared hypointense in the T1w image and hyperintense in the MDWI image after treatment.

Temperature changes were detected on the MR thermometry map during MRgFUS treatment, with all final peak temperatures exceeding 60°C (Figure 2). Sonication parameters and temperature measurements are listed in Table 1. Thermal dose distribution had contiguous appearance at the treatment site and was consistent with pre-treatment planning cell placement. All treatments showed an additional site of temperature increase seen in the sagittal view at the skull base inferior to the target region (Figure 2, top). After treatment, the target region appeared hypointense in the T1 image and hyperintense in the MDWI image (Figure 3).

### Histological Analysis and Lesion Volume

**Figure 4:**
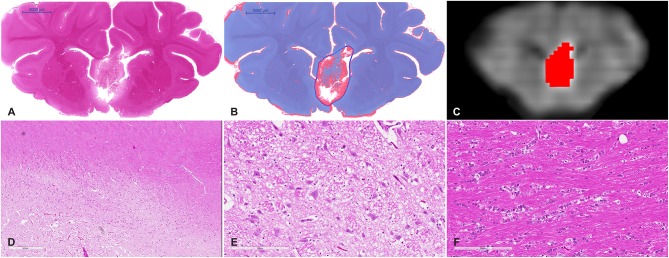
**(A)** Whole mount view of a coronal section of a representative MRgFUS treated piglet brain stained with H&E and **(B)** the same section after computer-based lesion segmentation (see methods). **(C)** A similar coronal view MDWI image of the brain overlaid with the lesion mask in red. **(D)** Medium magnification image showing the zone between destroyed tissue, vacuolated tissue, and preserved tissue (scale bar represents 600 μm). **(E)** High-magnification image of the treated region. Tissue is disrupted centrally and at the margins shows coarse parenchymal and perivascular vacuolation and vascular distention (scale bar represents 200 μm). **(F)** High magnification of untreated tissue showing preserved white matter (scale bar represents 200 μm).

The volume of the treatment sites from the histological data (including edema) and from the MDWI image are listed in Table 1. The values were comparable between the MDWI lesion volume and the lesion volume measured from the histological data including edema (paired *t* test: *P* > 0.05). Compared to the sonication cell volume of 83.78 mm^3^, the volume of the ablated tissue was roughly four times larger (range: 302–432 mm^3^). Figure 4 shows an example of the histological data after treatment. The similar coronal slice from T1 and MDWI images is also shown with the lesion boundary masked with red color. In the histological data, the region within the dark blue boundary line is considered as lesioned tissue based on slide color, intensity, background, and morphology. Substantial tissue disruption can be visualized.

Gross examination of coronal brain slices post-treatment revealed well-delineated lesions centered on the anterior body and column of the fornix. Microscopic evaluation of H&E-stained slides (Figure 4) showed a sharp area of coagulative necrosis with coarse parenchymal and perivascular vacuolation and vascular distention, in keeping with damage to the parenchyma. Signs associated with longer duration post-injury including nuclear karyorrhexis, cytoplasmic eosinophilia, and inflammatory cell infiltration were not observed at this early time point. Analysis of LFB-stained slices showed similar signs of coarse vacuolation and decreased stain contrast, indicating damage to white matter and myelin.

### Pre- and Post-MRgFUS Diffusion Metrics Comparison

**Figure 5:**
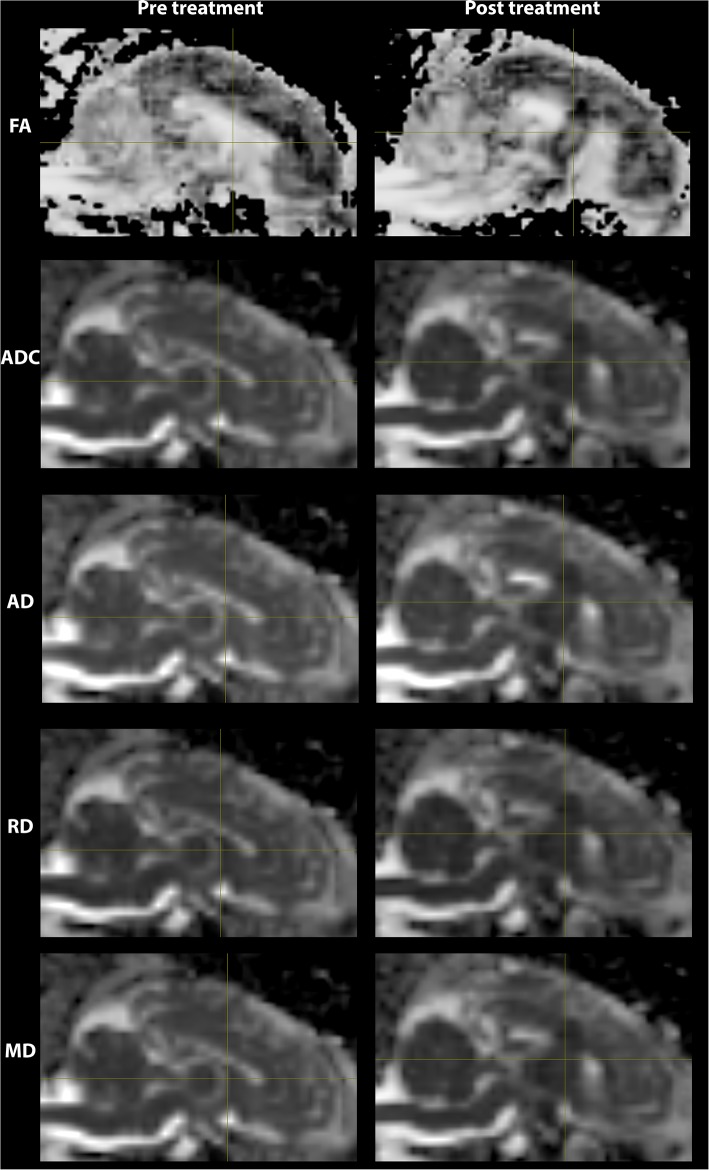
DWI metric maps (FA, ADC, AD, RD, and MD) before and after MRgFUS treatment for one subject. Hypointensity was present in all five metric maps after treatment.

**Figure 6:**
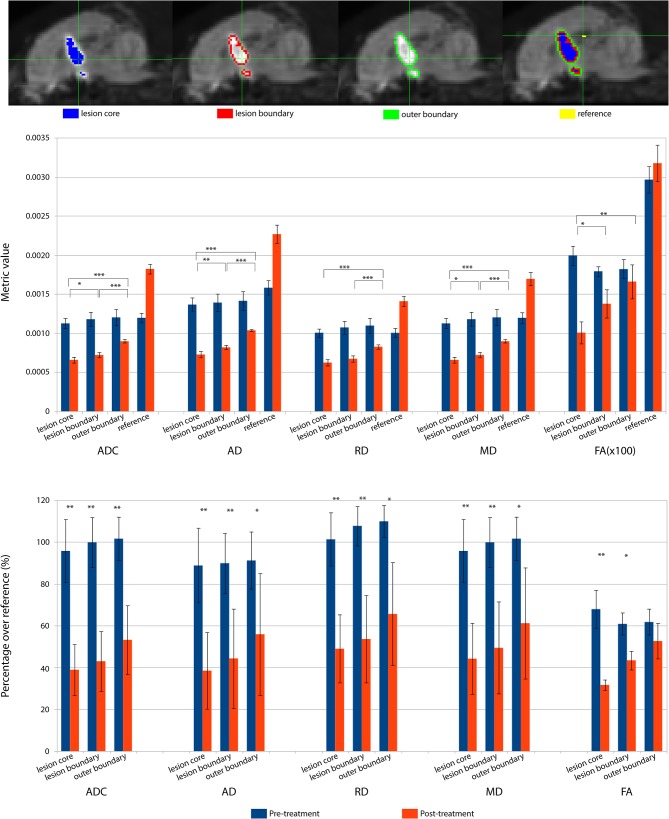
DWI metrics including ADC, AD, RD, MD, and FA in the lesion core, lesion boundary, outer boundary, and the reference region are shown pre- and post-treatment. **(Top)** Mid-sagittal slice of locations for different regions of interest (ROI). **(Middle)** Mean values within the ROIs for pre- and post-treatment. Asterisks represent the significant difference between the metric values in the lesion core, lesion boundary, and outer boundary after treatment, where *P < 0.05; **P < 0.01, ***P < 0.001. **(Bottom)** DWI metric values normalized over the value in the corresponding reference region, shown as percentage. Asterisks represent the significant difference between the metric values before and after treatment, where *P < 0.05; **P < 0.01, ***P < 0.001, FDR corrected.

The lesioned region was hypointense in all five metric maps of ADC, FA, AD, RD, and MD (Figure 5). In both raw value and normalized value over the reference region, all diffusion metrics were lower in the post-treatment tissue compared to the pre-treatment tissue in the lesion core, lesion boundary, and outer lesion boundary regions (Figure 6). Paired *t* test showed that the normalized values in the post-treatment regions were all significantly lower than in the corresponding region after treatment, except for a non-significant decrease in FA in the outer lesion boundary. The damage to the lesion core was the most severe, with decreasing severity with further distance from the core. ANOVA comparison showed significant differences between all three levels for raw metric values. The only exceptions were in RD between the lesion core and lesion boundary, and FA between lesion boundary and outer lesion boundary. While the outer lesion boundary presented smaller decreases in the pre- and post-treatment comparison, decreased metric values were persistent relative to pre-treatment measurements. Figure 6 shows the lesion in MDWI, with the three defined layers of lesion and the reference region in the splenium of corpus callosum (marked in yellow) for normalization. Both raw and normalized metric values in the different layers of the lesion region are shown. There was no significant difference in the DWI metrics between the three layers of tissue before treatment (*P* > 0.05).

### Pre- and Post-tractography Visualization

**Figure 7:**
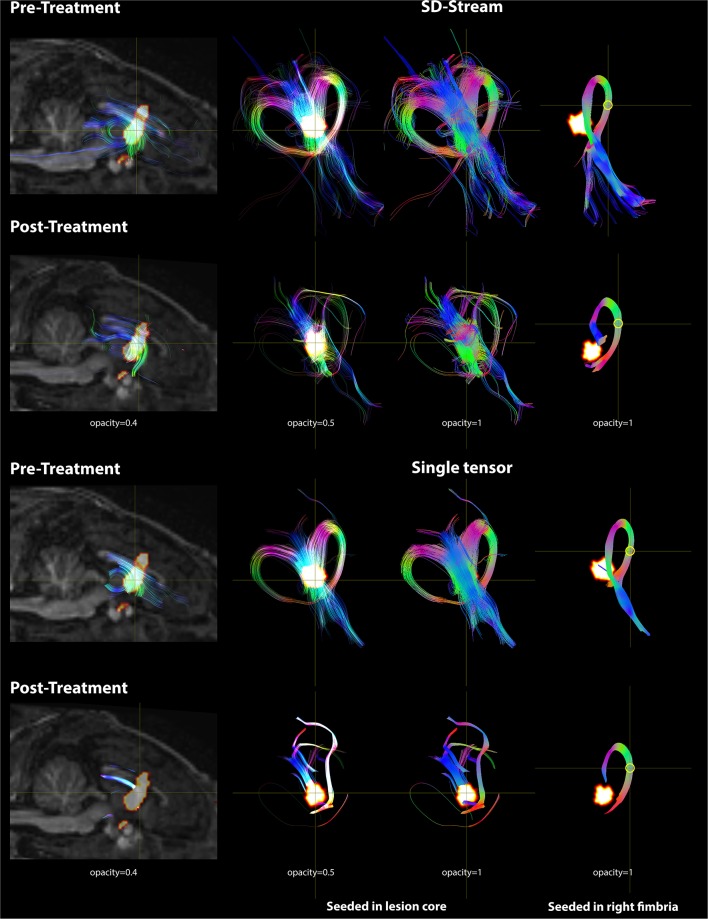
DWI tractography seeded in the lesion core and right fimbria is presented with both the FOD model using SD-Stream and the single tensor model. Left: mid-sagittal T1w image overlaid with the lesion core (seed region) and the tracts generated based on that seed (with opacity of 0.4). Middle: tracts seeded in the lesion core in 3D space with opacity of 0.5 and 1, respectively. Right: tracts seeded in the right fimbria (yellow circle) in 3D space. Tracts are colored by orientation (red, right–left; green, superior–inferior; blue, anterior–posterior).

Based on the FOD model, damage in the lesion core resulted to disorganized tract fragments in the body of the fornix and discontinuation of the tracts of the fimbria connecting to the body of the fornix. Based on the single-tensor model, no tracts were generated in the lesion core and tracts generated from the boundary of the lesion core did not appear to connect to the fimbria. When seeded in the right fimbria, the fiber tracts stopped at the lesion after treatment while they extended to the body of the fornix before treatment for both models. Tracts based on the FOD and single-tensor models generated for pre- and post-treatment data are shown in Figure 7.

## Discussion

MRgFUS is increasing in interest and application within neurosurgery. However, a thorough understanding of the effects of focused ultrasound and how it disrupts tissue architecture is needed. In this study, we use multimodal imaging, advanced diffusion imaging, and histology in an animal model to shed light on the effects of focused ultrasound on brain tissue and characterization of the histological profile of the lesion. We demonstrate that the clinical MRgFUS system can produce ablative lesions in the fornix of a piglet brain model, resulting in significant decreases in diffusion metrics (ADC, AD, RD, MD, and FA), and that MDWI is an effective semi-automated tool for identifying ablated brain tissue with histological data confirming the lesion volumes. Histological data successfully confirmed a sharp region of ablated tissue with displays of coarse vacuolation and vascular distention. Imaging in this acute phase did not show inflammation, suggesting that an additional, new volume of inflammation zone will develop subsequent to and surrounding the MRgFUS process. Fiber tracts passing through the lesions were observed with substantial disruption when seeded both within the lesion and distally.

The fornix was chosen as a model white matter structure to investigate the effects of non-invasive MRgFUS ablation and the ability of DWI to measure those effects. The impact of this treatment on white matter is not yet well-established. Most lesioning treatments are targeted at gray matter structures, namely, thalamic nuclei. However, there is growing interest in the use of MRgFUS to treat white matter directly including cingulotomy and capsulotomy for treatment-refractory obsessive compulsive disorder (8, 30, 31). The fornix specifically was chosen as a model to study white matter effects because it is a large central structure that is unambiguously located on anatomical T1-weighted images, accurately targeted by the transducer through the cranial window with minimal skull heating effects, and can be consistently reconstructed via tractography. The goal of this study was the DWI assessment of MRgFUS treatment and though tractography has the potential to enhance targeting of fiber pathways that are not easily detected with conventional MRI, tractography targeting guidance is beyond the scope of this work. However, the principles of tractography in terms of visualizing affected pathways may be readily applied to both gray and white matter structures. As demonstrated in this study, tracking seeds may be placed either within the lesion itself or in specific tracts from a location distal to the lesion in order to reconstruct fiber pathways relevant to the treatment. In a recent study of MRgFUS for essential tremor, we have employed the latter approach to visualize tract overlap with thalamotomy lesions in the presence of adverse side effects (26). This method is therefore relevant to treatments targeted at both white and gray matter structures, serving as a visual reference for treatment assessment and the extraction of DTI metrics for microstructural analysis.

We investigated the effect of peak temperature on lesion characteristics. A peak temperature of 60°C or higher was reached in all subjects, exceeding the therapeutic 55°C threshold for 100% necrosis in human brain tissue demonstrated by Jeanmonod et al. (7), in keeping with the histological data seen for our treatment area. It has been previously shown that therapeutic sonications above this threshold denature cellular proteins in the lesion area and can produce three observable concentric lesion zones on T2-weighted MRI of a necrotic center, apoptotic periphery, and surrounding edema (40, 41). Our study investigated the acute treatment effects (imaging <1 h after sonication), where T1 images revealed a hyperintense core surrounded by hypointense periphery, suggesting more than 1 h from treatment is required for all three distinct lesion zones to manifest in anatomical imaging. Our observed decreasing alterations in diffusion metrics with increasing radial distance from the lesion core supports the notion of a MRgFUS-induced necrotic lesion core surrounded by transient perilesional edema. This is further supported by our MR thermometry measurements, where temperature is highest in the treatment cell and decreasing radially, and the time–temperature relationship for thermal tissue damage (42).

Diffusivity metric changes including AD, RD, MD, ADC, and FA decreases are consistent with the substantial axonal damage observed in the histological data. Histology showed coagulative necrosis, coarse parenchymal and perivascular vacuolation, and vascular distention. Substantial disruption of cellular membranes and tissue integrity would be consistent with the decreased water movement and directionality observed in our diffusion measurements. While there is a lack of literature exploring the diffusivity and anisotropy changes after MRgFUS treatment of the brain, decreased ADC values after MRgFUS of uterine fibroids has been associated with restricted water flow due to loss of membrane integrity and cellular disruption (43). No signs of eosinophilia or inflammatory cell infiltration were observed at this early time point (<2 h after treatment), consistent with decreases in diffusivity in the lesion. We suspect that longer post-injury duration may lead to increases in RD, MD, and ADC as inflammatory cells flow into the lesion.

LFB staining, sensitive to myelin, showed coarse vacuolation, similar to H&E, and decreased stain contrast. This is consistent with observed decreases in RD, which is associated with myelin integrity. It has been shown previously that fat sustains greater damage compared to other tissues for a given thermal ultrasound dose (44). We thus speculate that myelin sheath fat content in white matter may display preferential absorption of thermal energy compared to other tissue types, such as gray matter, and result in preferential disruption to axonal tissue, consistent with our observed diffusion changes.

While changes of diffusion metrics in the treatment area confirm the effectiveness of MRgFUS treatment, MDWI also provides access to the volume of the area via high-contrast signal intensity with non-treated brain regions. MDWI lesion volume measurements are comparable to the volume estimated in histological data. Thus, MDWI may serve as a valuable tool for *in vivo* lesion volume estimation. MDWI segmentation is also a semi-automated process, dependent only on selecting a signal intensity threshold, thus avoiding the need for manual segmentation.

Since the lesion core estimated from the MDWI data may include both edema and necrosis, we investigated whether DWI metric changes in the lesion demonstrated a differential diffusivity metrics between edema and necrosis. We thus generated two areas of the lesion, the outer layer and inner core to calculate the DWI metric changes (Supplementary Figure 2). Comparison of these two regions showed that there was no significant difference in DWI metrics (two-sample *t* test, FDR correction, all *P* > 0.05). One explanation is that there might be partial volume effects distorting the metric values of neighboring voxels in the lesion. Another reason might be that splitting the two layers based on the lesion shape may not reflect the real damage pattern on the treatment cell. However, the temperature map obtained during the MRgFUS treatment was not a continuous 3D image, which thus limited our application of it on lesion segmentation. Future work on a robust method to segment the lesions is still needed.

After treatment, fiber tracts seeded from the core region of the lesion were disrupted. Only voxels on the boundary of the ROI showed connections to the bilateral fimbria. This may reflect severe damage in the center of the seed (necrosis) and less severe damage (edema) on the boundary. The tracts generated from inside of the seed region presented in short segments without continuing to the outside of the seed region. When seeded in the right fimbria, the tracts stopped in front of the lesion without connecting to the body and column of the fornix. This is consistent with previous tractography findings in lesioned tissue where distally seeded tracts did not pass through the lesion (45–47). A similar approach of tracking from specific pathways of interest has shown improved accuracy in targeting of thalamic nuclei and determination of perimeters of safe dosage with respect to neighboring pathways (27–29). A recent study revealed a relationship between the presence of adverse MRgFUS treatment effects and observations of lesion overlap with tracts responsible for the functions impaired by off-target lesioning (26). These studies and our results suggest that seeding from within the lesion may present a method of visualizing all tracts affected by the treatment while seeding distally may reveal the extent of damage to a specific pathway of interest.

We showed that based on the current acoustic power and sonication strategy, the ablated tissue volume was larger than our focal point. This was expected as the estimated focal cell size does not account for thermal diffusion and conductivity. There was also heating at the base of the skull in the far field region. This could be due to the proximity of the skull base in this small animal model or the high acoustic attenuation of bone compared to brain tissue.

Our current experimental setting provides guidance for future experiment. Previous studies have measured MRgFUS lesion evolution at longer time points; however, they did not incorporate DWI and thus their *in vivo* microstructural assessment is limited (40, 48). Future work will focus on a lower power regime to limit off-target thermal dose deposition and induce less disruption than observed in this study for a more nuanced interpretation of diffusion metric changes in the brain tissue after treatment. Lower power treatments would also apply readily to survival studies in order to assess the longitudinal impact of MRgFUS on brain diffusion parameters.

## Conclusion

We have demonstrated the utility of DWI in the identification of brain lesions and assessment of the efficacy of MRgFUS treatment. Given the observations in this study, diffusion imaging has the potential to advance MRgFUS through *in vivo* microstructural lesion and fiber tractography assessment.

## Data Availability Statement

The datasets generated for this study are available on request to the corresponding author.

## Ethics Statement

The animal study was reviewed and approved by Animal Care Committee and Laboratory Animal Services at the Hospital for Sick Children, Toronto ON, Canada.

## Author Contributions

All authors contributed to this work. The whole research team participated in the experimental design and analysis. Data were collected by MW, JZ, AW, TL, and KP. The manuscript was written by MW and JZ. Manuscript critique and interpretation were contributed by AW, KP, CH, JD, and MH.

### Conflict of Interest

The authors declare that the research was conducted in the absence of any commercial or financial relationships that could be construed as a potential conflict of interest.
