# Supplementary figures and images for: Acute MR-Guided High-Intensity Focused Ultrasound Lesion Assessment Using Diffusion-Weighted Imaging and Histological Analysis
Source: Front Neurol. 2019 Oct 15;10:1069. doi: 10.3389/fneur.2019.01069 (PMC6803785; doi:10.3389/fneur.2019.01069)

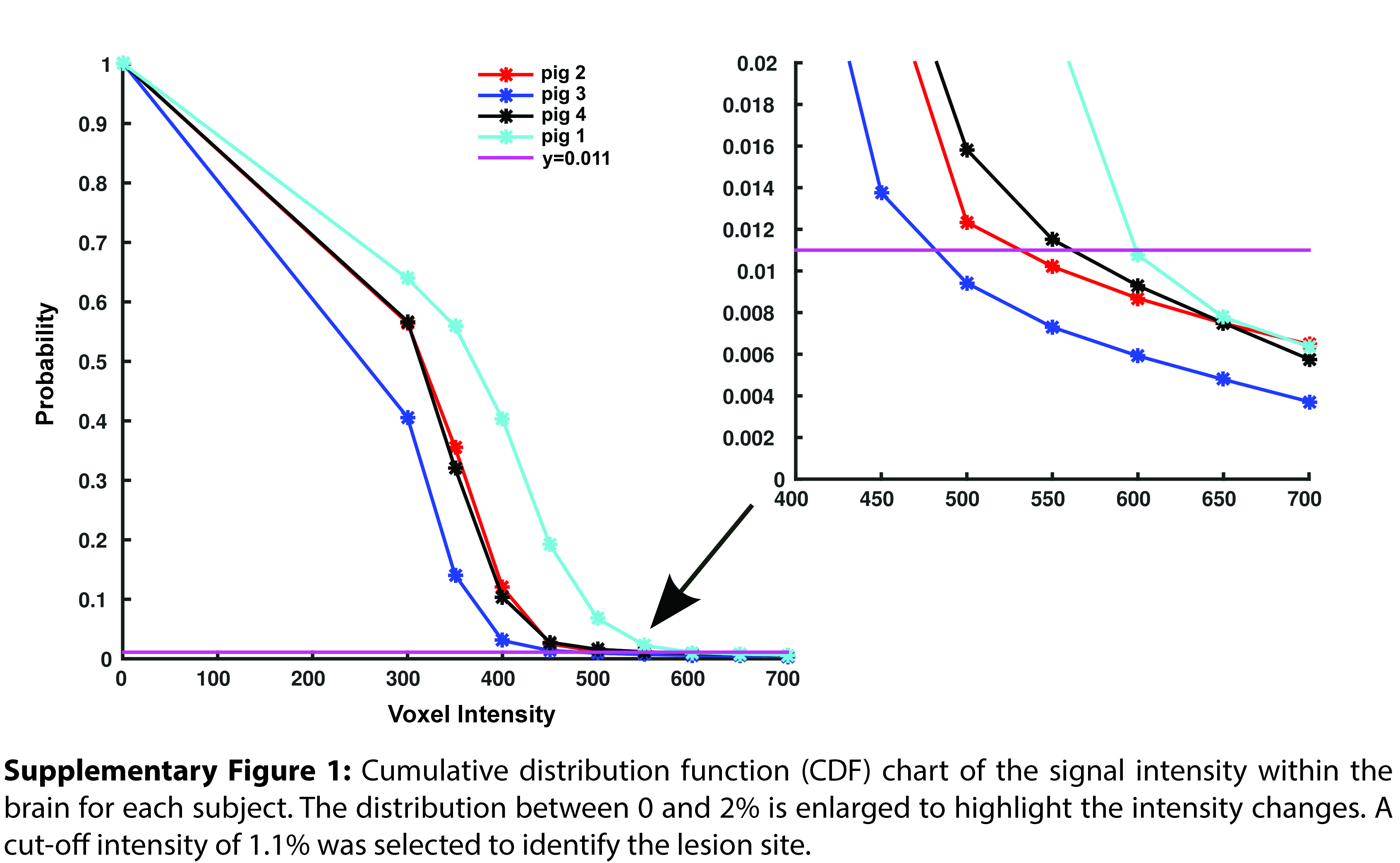

Supplement: Supplementary file 1 [file Image_1.TIF]

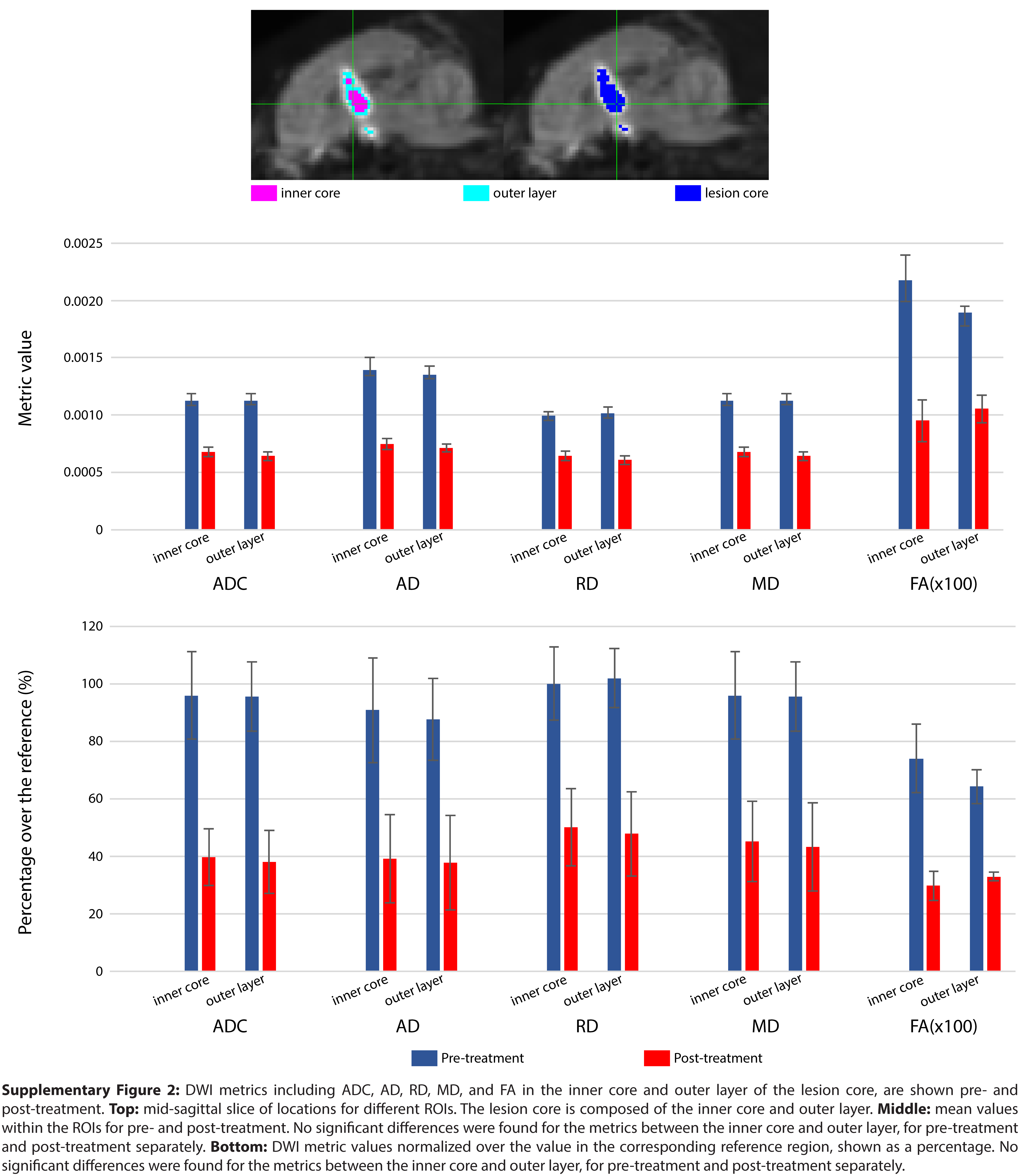

Supplement: Supplementary file 2 [file Image_2.TIF]
